# Detection and analysis of COVID-19 in medical images using deep learning techniques

**DOI:** 10.1038/s41598-021-99015-3

**Published:** 2021-10-04

**Authors:** Dandi Yang, Cristhian Martinez, Lara Visuña, Hardev Khandhar, Chintan Bhatt, Jesus Carretero

**Affiliations:** 1 Beijing Electro-Mechanical Engineering Institute, Beijing, 100074 China; 2 Department of Computer Science and Engineering, Carlos III University of Madrid, 28911 Madrid, Spain; 3 U & P U. Patel Department of Computer Engineering, CSPIT, Charotar University of Science and Technology (CHARUSAT), Changa, India

**Keywords:** Respiratory tract diseases, Imaging, Computer science

## Abstract

The main purpose of this work is to investigate and compare several deep learning enhanced techniques applied to X-ray and CT-scan medical images for the detection of COVID-19. In this paper, we used four powerful pre-trained CNN models, VGG16, DenseNet121, ResNet50,and ResNet152, for the COVID-19 CT-scan binary classification task. The proposed Fast.AI ResNet framework was designed to find out the best architecture, pre-processing, and training parameters for the models largely automatically. The accuracy and F1-score were both above 96% in the diagnosis of COVID-19 using CT-scan images. In addition, we applied transfer learning techniques to overcome the insufficient data and to improve the training time. The binary and multi-class classification of X-ray images tasks were performed by utilizing enhanced VGG16 deep transfer learning architecture. High accuracy of 99% was achieved by enhanced VGG16 in the detection of X-ray images from COVID-19 and pneumonia. The accuracy and validity of the algorithms were assessed on X-ray and CT-scan well-known public datasets. The proposed methods have better results for COVID-19 diagnosis than other related in literature. In our opinion, our work can help virologists and radiologists to make a better and faster diagnosis in the struggle against the outbreak of COVID-19.

## Introduction

COVID-19 is the disease caused by the coronavirus called SARS-CoV-2. COVID-19 is the name given by the World Health Organization (WHO) on February 11, 2020 (World Health Organization, 2020)^1^. Since the discovery of the first case, the disease has spread to almost every country, causing deaths of over 4 million people among nearly 180 million confirmed cases based on the statistics of the World Health Organization by June 2021^2^.

The first step in the treatment of COVID-19 is to screen patients in primary health centers or hospitals. Although the final diagnosis still relies mainly on transcription-polymerase chain reaction (PCR) tests, in case of people with strong respiratory symptoms the election protocol nowadays in hospitals relays on medical imaging, as it is simple and fast, thus helping doctors to identify diseases and their effects more quickly^3^. Following this protocol, patients that are suspected to suffer COVID-19 undergoes first an X-Ray session and then, in the case that more details are needed, they take a CT-scan session. As a result of this protocol, computed tomography scan (CT scan) and X-ray images are being widely used on the clinic as alternative diagnostic tools for detecting COVID-19 and to find the effects of the virus^4^.

To make the diagnosis, doctors visualize the lungs on X-ray or CT-scan images and search for symptoms of COVID-19 deformation. The high transmission rate of COVID-19 has resulted in a large influx of patients into hospitals in a short period of time, placing a significant burden on imaging physicians and often resulting in doctors’ shortages of the fight against the disease. This problem can be solved by using deep learning methods, which have continued to make significant progress in recent years, mainly due to the increasing computing power and the continuously growing amount of available data, as well as the continuous improvement of deep learning models and their algorithms, as demonstrated in challenge competitions to achieve record-breaking performances^5^. The essence of deep learning is to learn more accurate features by building a multi-hidden layer machine learning model that is trained with a large amount of sample data to eventually improve the accuracy of classification or prediction^6,7^.

A great deal of work has been paid to learning in medical images analysis^8,9^. However, the analysis still requires expertise and includes a variety of algorithms to enhance, speed up and render an accurate diagnosis^10,11^. Deep learning algorithms have achieved better efficiency in pneumonia detection and demonstrated high precision compared to previous approaches^12,13^. However, currently, in hospitals doctors may find patients with pneumonia caused by flu, other viruses, and COVID-19 at the same time. Thus, there is a need for a fast and accurate method of detection that can identify both kinds of pneumonia.

Recently, COVID-19 pneumonia detection approaches based on deep learning have been described by several groups^14–16^. Alshazly^14^ used deep CNN architectures on CT-scan images to detect COVID-19 with accuracy, precision, and sensitivity of 93.96%, 99.13% and 94% respectively. Ayrton^15^ presented ResNet50 based deep transfer learning technique and reported the validation accuracy of 96.2% with a small dataset of 339 images for training and testing.Wang^16^proposed five pre-trained deep learning models, which the Xception model showed a relatively ideal effect, and the accuracy reached 96.75%. The dataset contains 1102 chest X-ray images of healthy patients and COVID-19 positive patients, randomly divided into the training set and test set. Therefore, advancing deep learning to detect and diagnose the lung medical images of patients with new COVID-19 pneumonia is needed.

This demand has motivated us to write this article to conduct a comparative analysis of the state-of-the-art methods for COVID-19 X-ray and CT scan images classification, to research the structure of deep neural networks used in this field, to analyze the advantages and disadvantages of them, and to test the most suitable ones by using enhanced datasets build from public repositories.

The main contribution of this paper is as follows:

- The validity of the algorithms was evaluated using our proposed framework on three well-known X-ray and CT-scan image public datasets.
- Apply transfer learning, which is adopted to overcome the overfitting problem caused by the limited number of training images in deep learning. Owing to the lack of a public COVID-19 dataset, we prepared a dataset containing 3616 chest X-ray images of COVID-19 positive patients.
- The proposed Fast.AI framework compared with previous works in terms of several performance metrics such as accuracy, f1-score, precision, and recall. All of the metrics have improved significantly.
- With an extensive evaluation to validate the proposed methods, we find the proposed VGG16 deep transfer learning model shows excellent performance on binary and three-class classification tasks, the accuracy of the best model is as high as 99%.

This paper is organized as follows. In section “Methods” we present our proposed CNN models, the datasets used in this paper, the evaluation metrics and the experimental setup for each analysis performed. In section “Experimental results”, presents the results of X-ray images and CT-scans images obtained with our proposed models. We compare our results with the state-of-the-art methods in the section “Discussion”. Finally, main conclusions are provided in section “Conclusion”.

## Methods

All methods were carried out in accordance with relevant guidelines and regulations.

### Models proposed

#### Transfer learning, Fast.AI, CNN architectures

**Figure 1:**
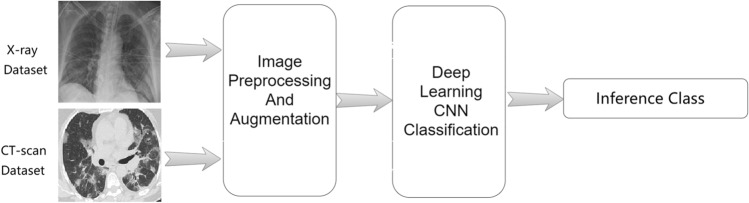
Deep learning based screening structure of COVID-19.

Several deep learning networks have been used to diagnose COVID-19 effectively^17–19^. Among them, CNN is the main technique for classification, segmentation, and prediction of COVID-19 disease. In Fig. 1 we introduce a COVID-19 deep learning-based screening structure, where the program uses a deep learning algorithm to predict whether the images of the suspected lung of the patient are normal, have bacterial pneumonia, or COVID-19.

As part of this work, we have used deep learning to train X-ray and CT-scan images separately. COVID-19 X-ray binary and multi-class classification are performed by utilizing enhanced VGG16 deep transfer learning models, the model performance shows promising results and is simple to implement. On the other hand, we used four pre-trained CNN models, VGG16, DenseNet121, ResNet50, and ResNet152, for COVID-19 CT-scan image binary classification, and proposed the Fast.AI ResNet framework in the detection of COVID-19 CT-scan images with high accuracy.

**Figure 2:**
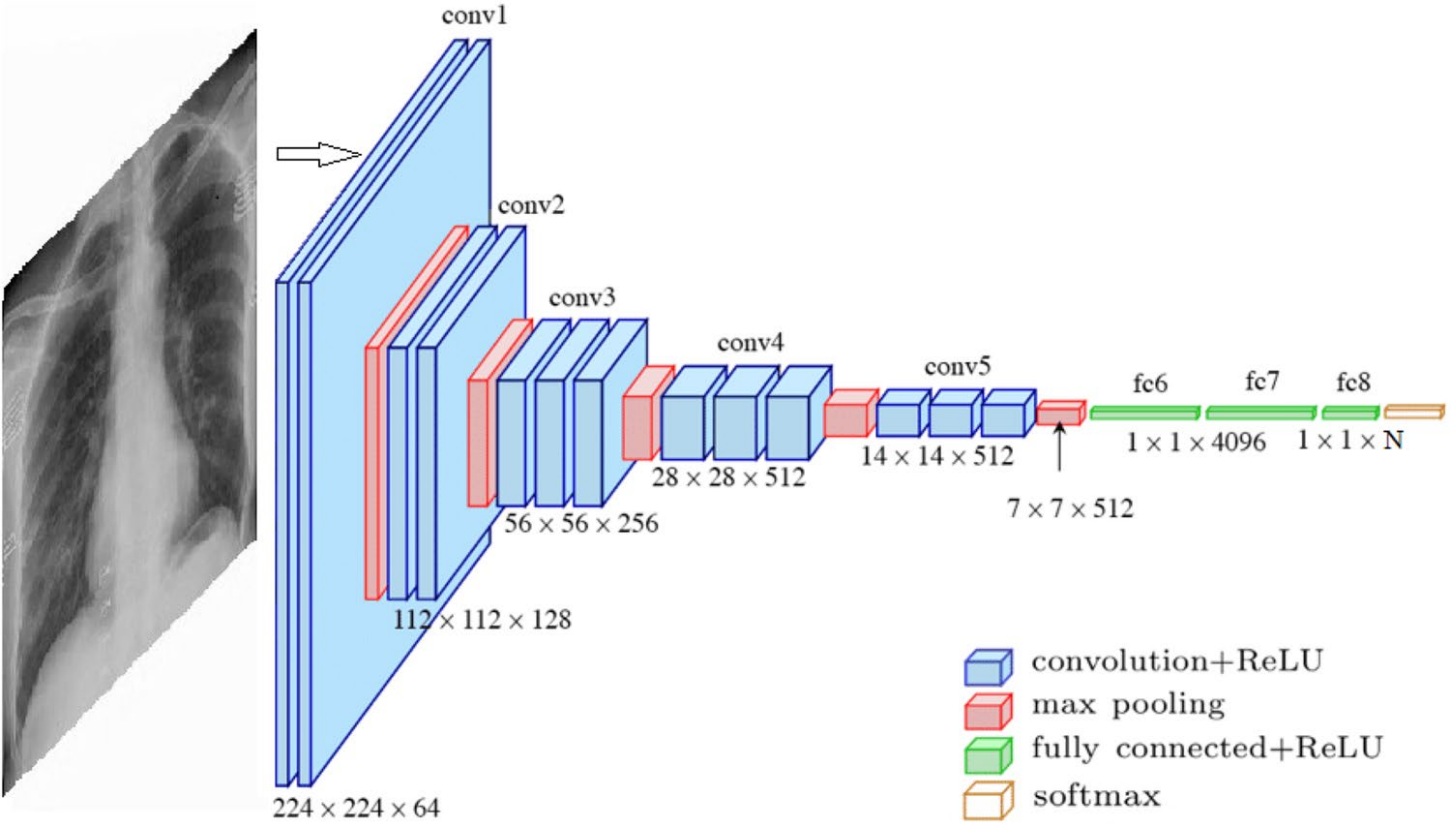
The structure of VGG16 model.This figure was created with Image online.co and exported under a free subscription.

**Figure 3:**
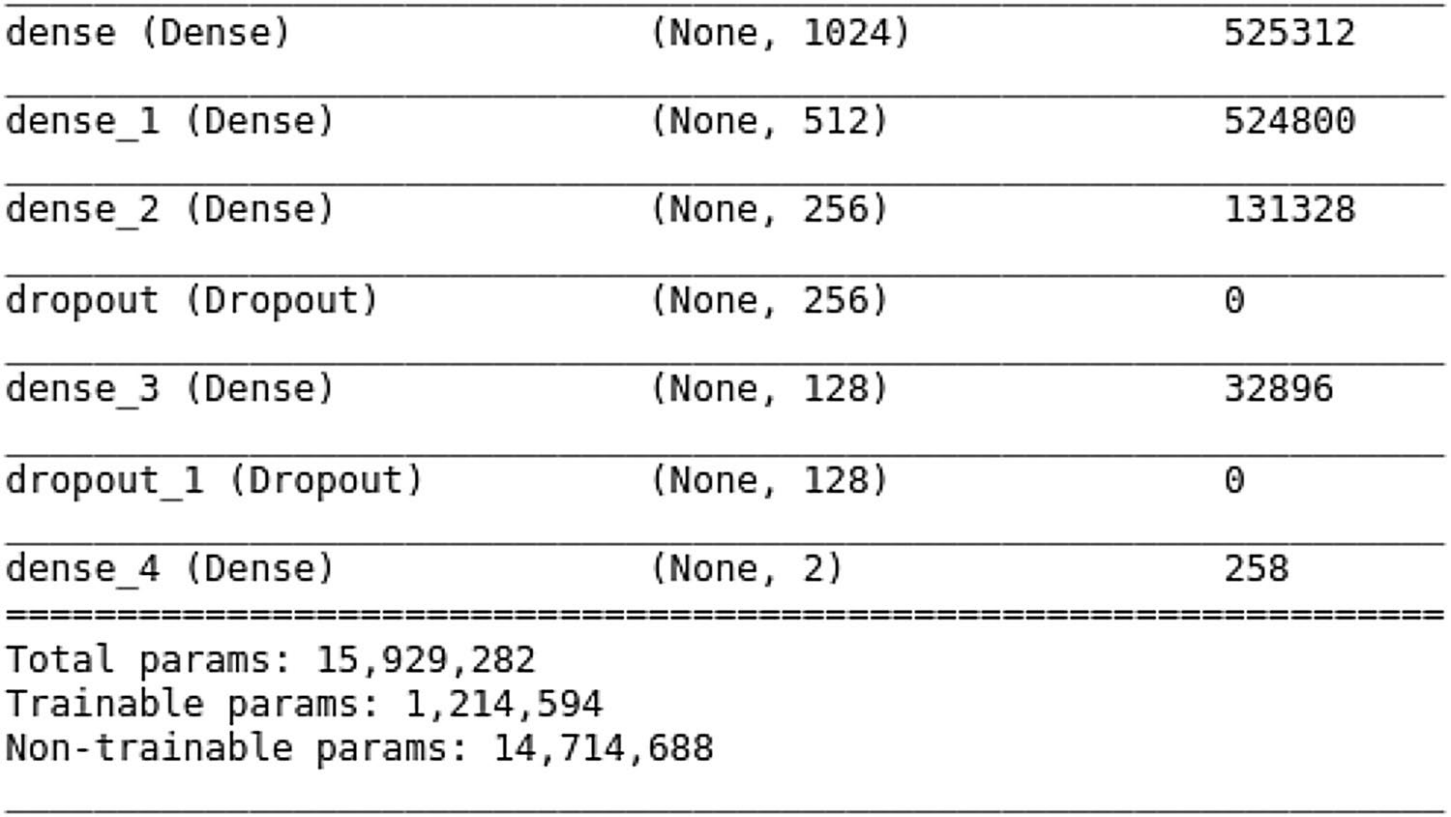
Fine-tuning based on VGG16 pre-training.

**Figure 4:**
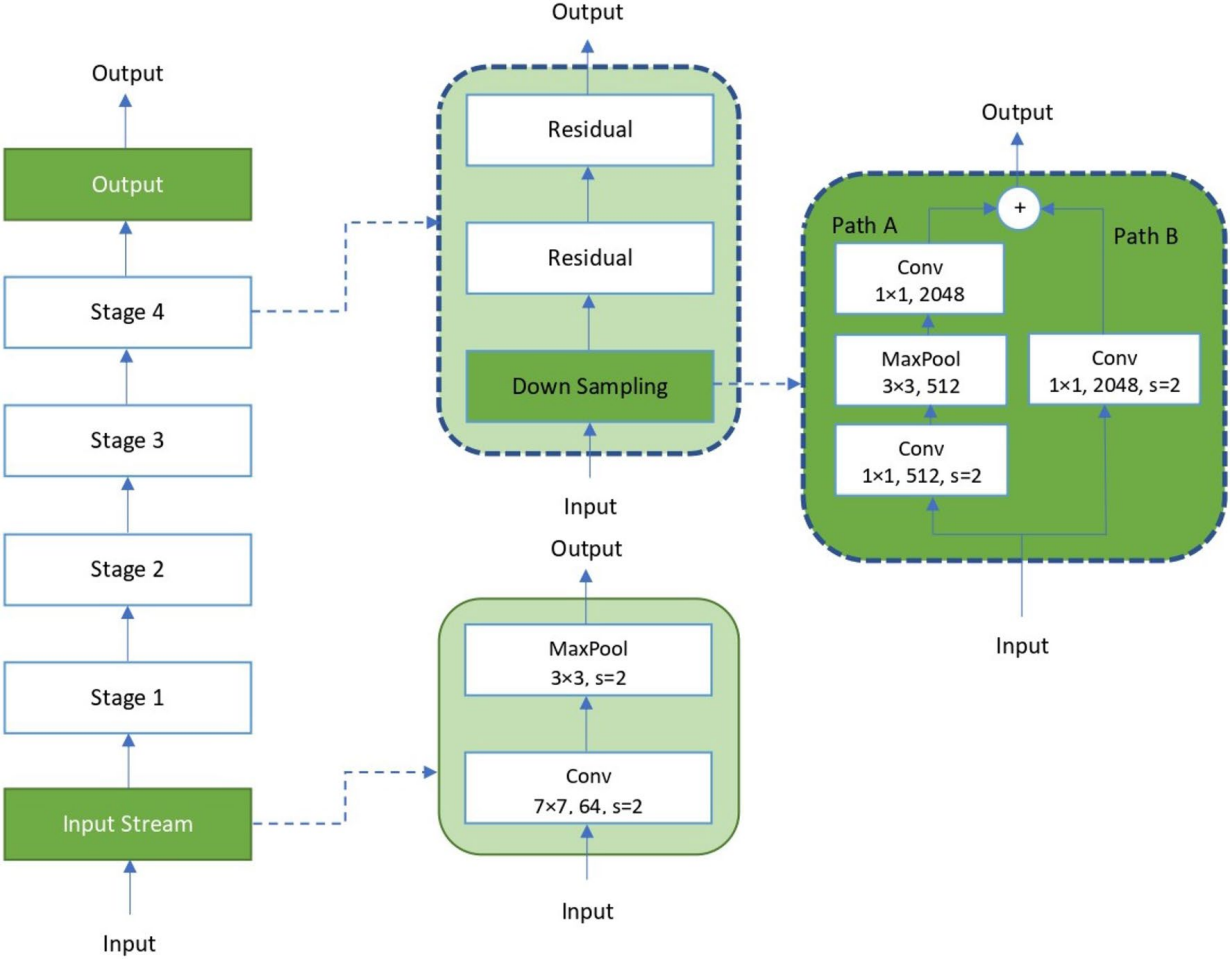
The ResNet Architecture, the convolution kernel size, output channel size and stride size (default is 1) are illustrated, similar for pooling layers.This figure was created with Microsoft Paint.

- VGG16: VGG16 is a CNN architecture that, despite having been developed in 2014, is still considered today to be one of the best architectures for image classification^20^. As shown in Fig. 2, the VGG16 network consists of 16 layers, where convolutional layers (13) with $3 \times 3$ filters and $2 \times 2$ max-pooling layers are stacked. Between these layers, the relu activation function is applied. Then, there are three fully connected layers that contain most of the parameters of the network. Finally, a softmax function is used to produce the probabilities for each classification of pulmonary symptoms^21^. The VGG16 model is a successful use of convolutional neural networks in image recognition algorithms as the basic network. It has a specific network structure that is simple to change.
- Transfer Learning: Additionally, we applied a transfer learning technique by using ImageNet data onto application for comparatively smaller dataset. It reduces the long training period required by deep learning algorithms^22^. The model trained on ImageNet has been published, and other data sets can be fine-tuned using it. Since it adapts well to other data sets, it’s simple to apply the transfer learning application to the tagged data set in the specific issue scenario. In this work, our proposed VGG16 model is trained during 20 epochs and using a batch size of 32. In each epoch, every image is randomly modified with the ImageDataGenerator of Keras. We selected the adam optimizer from Keras with the learning rate of 0.001.The network uses a softmax classifier for binary classification. The fine-tuned pre-trained model with layers is used for feature extraction. In Dense Layer, the base neural networks have been frozen to preserve ImageNet weights during the training phase. Dropout is applied in the fully connected layers, to avoid overfitting in the model. It is typically taken as about 0.5,and the model is trained to get some metrics. If the overfitting is significantly better, but the metrics also drop significantly, try to reduce the dropout. If the overfitting is still severe, increase the dropout. For our case, the drop ratio of 0.3 and 0.2 have been used. Figure 3 shows fine-tuning based on VGG16 pre-training.
- ResNet: ResNet Architecture^23^ consists of an input layer, 4 ensuing stages and an output layer, as shown in Fig. 4. Each stage represents a part of the process we are executing consecutively. It receives input from previous stages, executes one step of the CNN, and provides the output. ResNet is divided into 5 stages, where the structure of Stage 0 can be regarded as a pre-processing of INPUT, and the last 4 Stages are composed of a Bottleneck and have a more similar structure. We have an input stem that performs a 7–7 convolution, has an output channel of 64, and a stride of 2. Next, we have a 3–3 max pooling-layer, with a stride of 2. In this layer, we are effectively decreasing 4 times the input width and height, and we increase the channel size to 64. On stage 2, and the subsequent ones, we have a down-sampling block and residual blocks. The residual blocks function in the same manner as the down-sampling one, the only difference would lie in the stride of the convolutions, which in this case would be 1. Changing the number of residual blocks we obtain different models, thus with ResNet50 and ResNet152 we are just indicating the number of the convolutional layers we have in the network.
- Fast.AI: Fast.AI is a deep learning library which provides practitioners with high-level components that can quickly and easily provide state-of-the-art results in standard deep learning domains and provides researchers with low-level components that can be mixed and matched to build new approaches^24^. It offers a great deal of features as well as functionality that makes developers customize the high-level API without getting involved with low-level API parts. Fast.AI provides new functionality around our neural network, such as visualization methods for data, more ways for loading and splitting data, inferring the number of classes from the dataset we provide, and extension of the training utilities by a concept called “callbacks”. For this paper We adopted the Datablock customization that allowed us to load the data in a more easy and structured way.

### Datasets

#### X-ray images datasets

We used a combination of two datasets to conduct the experiments with the VGG16 model. They include scanned chest X-ray images with classes (COVID-19, Normal) and (COVID-19, Pneumonia) respectively. These images are pre-processed and used for training the models of the CNN.

The first dataset entitled “COVID-19 Radiography Database”^25^ currently includes hundreds of X-rays images. The researchers from Qatar University, along with their collaborators from Pakistan and Malaysia, in collaboration with medical doctors have created a database of chest X-ray images for COVID-19 positive cases along with Normal and Viral Pneumonia images. They released 3616 COVID-19 positive cases along with 10,192 Normal, 6012 Lung Opacity and 1345 Viral Pneumonia images. The second data set entitled “Labeled Optical Coherence Tomography and Chest X-Ray Images for Classification”^26^ contains images (chest radiography) that are classified as normal and with some type of pneumonia.

We used two databases to create our data set, the collected dataset consists of 8461 total chest X-ray images including 3616 COVID-19, 1345 Pneumonia, and 3500 Normal. The collected dataset is divided into three parts. The training set is used for model training and learning as well as adjusting the parameters. The validation set is used to test the model in the training, optimize the model, and fine-tune the model parameters. Test sets are used to prove the final results of our model. First, we chose 1692 (20%) of the images of each class for testing, the remaining 6769 (80%) was randomly split again into training and validation splits (75–25%). All the images in the database are CRX in PNG format and a size 256 $\times$ 256.

#### CT-scan images dataset

We have used the dataset entitled “SARS-CoV-2 CT Scan Dataset”^27^, which is possibly the largest publicly available dataset of CT scans for COVID-19 identification. The dataset contains 1252 CT-scans that are diagnosed positive for the SARS-Cov-2 infection and 1229 CT-scans for normal healthy patients that are non-infected, comprising a total of 2481 CT-scan images. This entire data has been collected from actual patients in the hospitals from Sao Paulo in Brazil. The dataset aims to encourage the research and development of artificial intelligence-enabled methods to identify whether a patient is infected by the deadly virus through the analysis of his/her scan.

Owing to the inconsistent number of X-ray and CT-scan images, we want to verify the effectiveness of proposed model trained for different scales of images. We divided CT-scan dataset into 1737 images for training(70% )and 744 images for validation(30%), which is set of COVID, Non-COVID.

#### Performance evaluation metrics

To assess the classification of COVID patients, we run the models described in the previous section using the datasets presented for X-ray and CT-Scan images, adjusting the models in the training process to enhance their accuracy.

For each model we present three results that are typical in CNNs:

- Model accuracy curve.
- Model loss curve.
- Confusion matrix.

The model accuracy curve for training and validation, to show how well the model is training/generalizing. The gap between the training and validation accuracy indicates the amount of overfitting. The model loss curve gives a snapshot of the training process and the direction in which the network learns, A large gap between the training and validation curves indicates that the network can still learn more with training. A confusion matrix is a table that is used to describe the performance of a classifier in a set of test data for which we already know the true values. There are four basic terms associated with every confusion matrix^26^. (i) True Positives [TP]: These are the cases in which we predicted “yes” and patients do have the disease. (ii) True Negatives [TN]: We predicted “no” and they don’t have the disease. (iii) False Positives [FP]: We predict “yes” for the disease, but the patients don’t actually have the disease. This is also known as Type I error. (iv) False Negatives [FN]: Our model predicts “no” but patients have the disease. This is termed as Type II error. It is used to visualize important predictive analytics, which makes easier to understand and get relevant trends of the experiment.

Several traditional measurements were used to assess performance, four metrics are used here to evaluate models: Accuracy (ACC) , Precision (P) , Recall (R) , F1-score(F1).

1 $$\begin{aligned} ACC=  \dfrac{TP+TN}{TP+TN+FP+FN} \end{aligned}$$

2 $$\begin{aligned} P=  \dfrac{TP}{TP + FP} \end{aligned}$$

3 $$\begin{aligned} R=  \dfrac{TP}{TP + FN} \end{aligned}$$

4 $$\begin{aligned} F1=  2 \times \frac{(P\times R)}{(P+R)} \end{aligned}$$

### Experimental setup

The experimental part with the VGG16 model was carried out in three scenarios. In the first scenario, a classification model was developed containing images of normal patients together with patients identified with COVID-19. In the second scenario, we developed a model was developed to discriminate COVID-19 from Pneumonia (viral or bacterial) in those patients with abnormal X-ray images.In the third scenario, we considered three classes,which are COVID-19,Normal and Pneumonia.

These three scenarios were implemented using the Python programming language. To train the models, tools, libraries, and resources of TensorFlow 2.0 (with Keras), we used an open-source deep learning framework. All the required software was encapsulated using the Docker platform for the purpose of reproducing the experiments. Additionally, the necessary libraries (e.g. CUDA 10.2) to run each model on GPUs were included and encapsulated in the container. The GPU specification used for this experiment was an NVIDIA GeForce GTX 1060 with 6GB/PCIe/SSE2, Intel Core i5-3570 CPU 3.40 GHz—4, 15.3 GiB RAM and 3.0 TB.

For the CT-scan images experiments we used Fast.AI ResNet50 and Fast.AI ResNet152 models. They were run on Google Colaboratory with enhanced GPU. The GPU specification used for this experiment was Tesla K80 with 12GB of GDDR5 VRAM, Intel Xeon Processor with two cores at 2.20 GHz and 13 GB RAM.

### Ethical approval

All the data was obtained from publicly available datasets, each complying with the ethical standards of the respective institutional and/or national research committees, as well as with the Helsinki Declaration and its later amendments or comparable ethical standards.

## Experimental results

### X-ray images results for COVID-19/normal

Table 1 shows the results of the enhanced VGG16 model for the classification of COVID-19/normal on the validation and test data. The test images have never been used to train or tune hyperparameters, the performance is even better than the validation images, achieving up to 98% accuracy, 99% Recall (AKA Sensitivity), 98% Precision and 98.8% F1-score for our model.

**Table 1:** Classification performance obtained by enhanced VGG16 on X-ray images: COVID-normal.

COVID-19/normal	Accuracy (%)	Precision (%)	Recall (%)	F1-score (%)
Validation data	97	98	95	96.5
Test data	98	98	99	98.5

**Figure 5:**
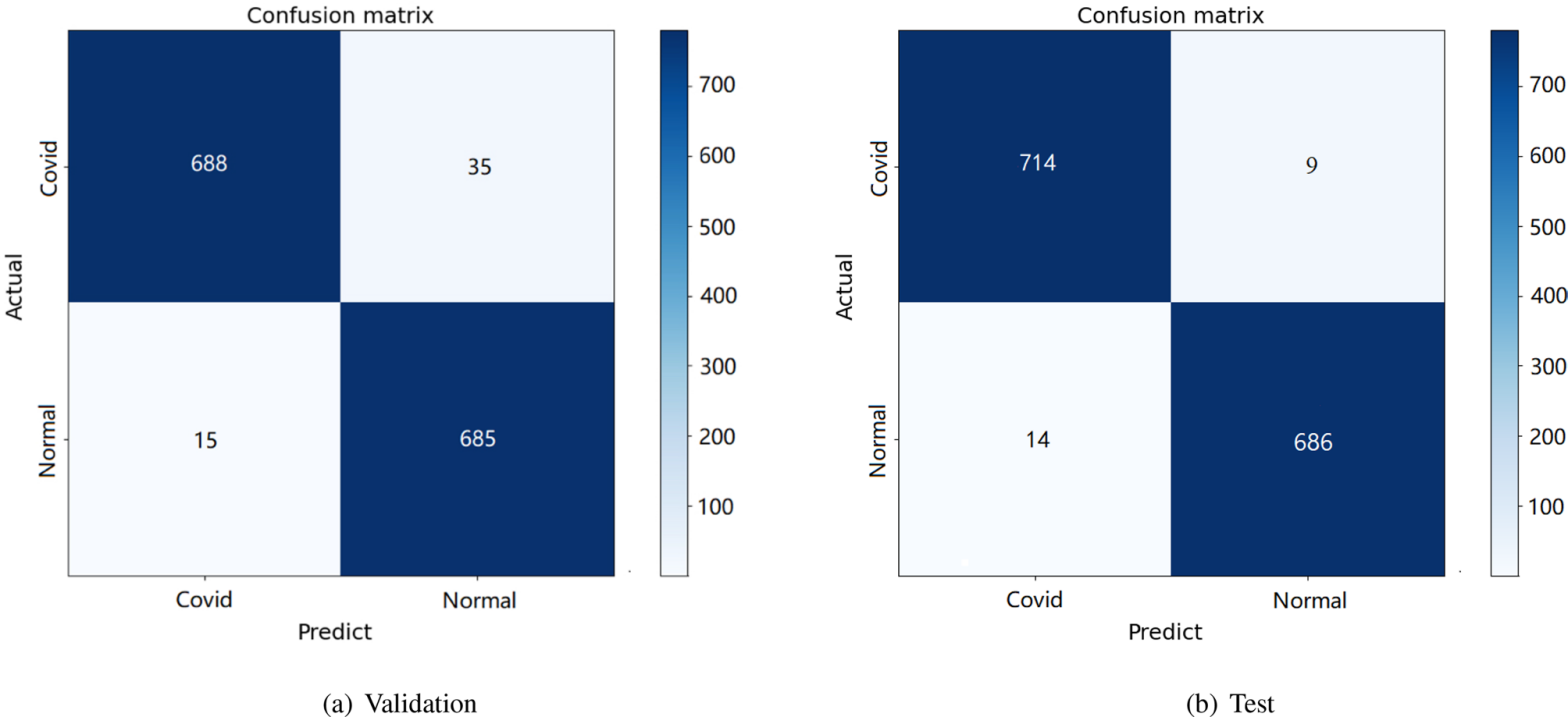
Confusion matrix for VGG16 on X-ray dataset: COVID-normal.

**Figure 6:**
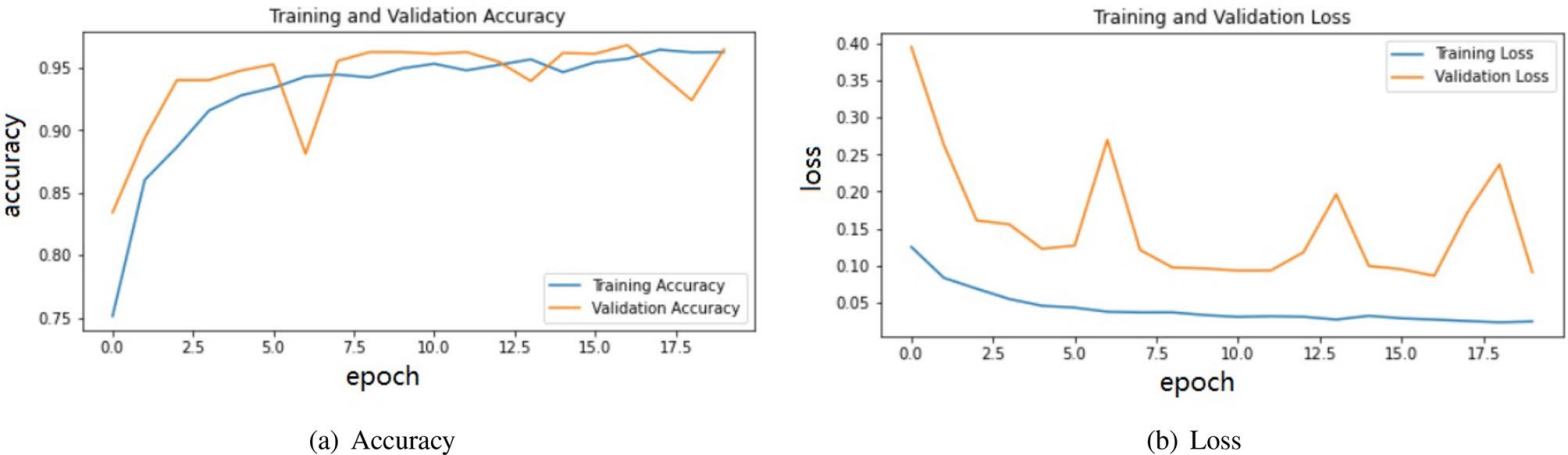
Model VGG16 for COVID/normal on X-ray dataset.

Figure 5 shows the confusion matrix of validation and test data for two-class classification with COVID-19/normal. The Fig. 6 shows the training and validation accuracy and loss for the VGG16 model.As can be seen, since the gap between the train and validation curves is minimum, the model converges well.

### X-ray images results for COVID-19/pneumonia

Table 2 shows the results of the enhanced VGG16 model for the classification of and COVID-19/pneumonia on the validation and test data. As may be seen, the result of validation and test data are both correctly made with a very high accuracy, 99%, recall (AKA Sensitivity) and Precision are also above 99%. Those results are significantly better than other studies in the literature, as we show in “Discussion”.

**Table 2:** Classification performance obtained by enhanced VGG16 on X-ray images: COVID-19/pneumonia.

COVID-19/pneumonia	Accuracy (%)	Precision (%)	Recall (%)	F1-score (%)
Validation data	99	100	99	99
Test data	99	99	99	99

**Figure 7:**
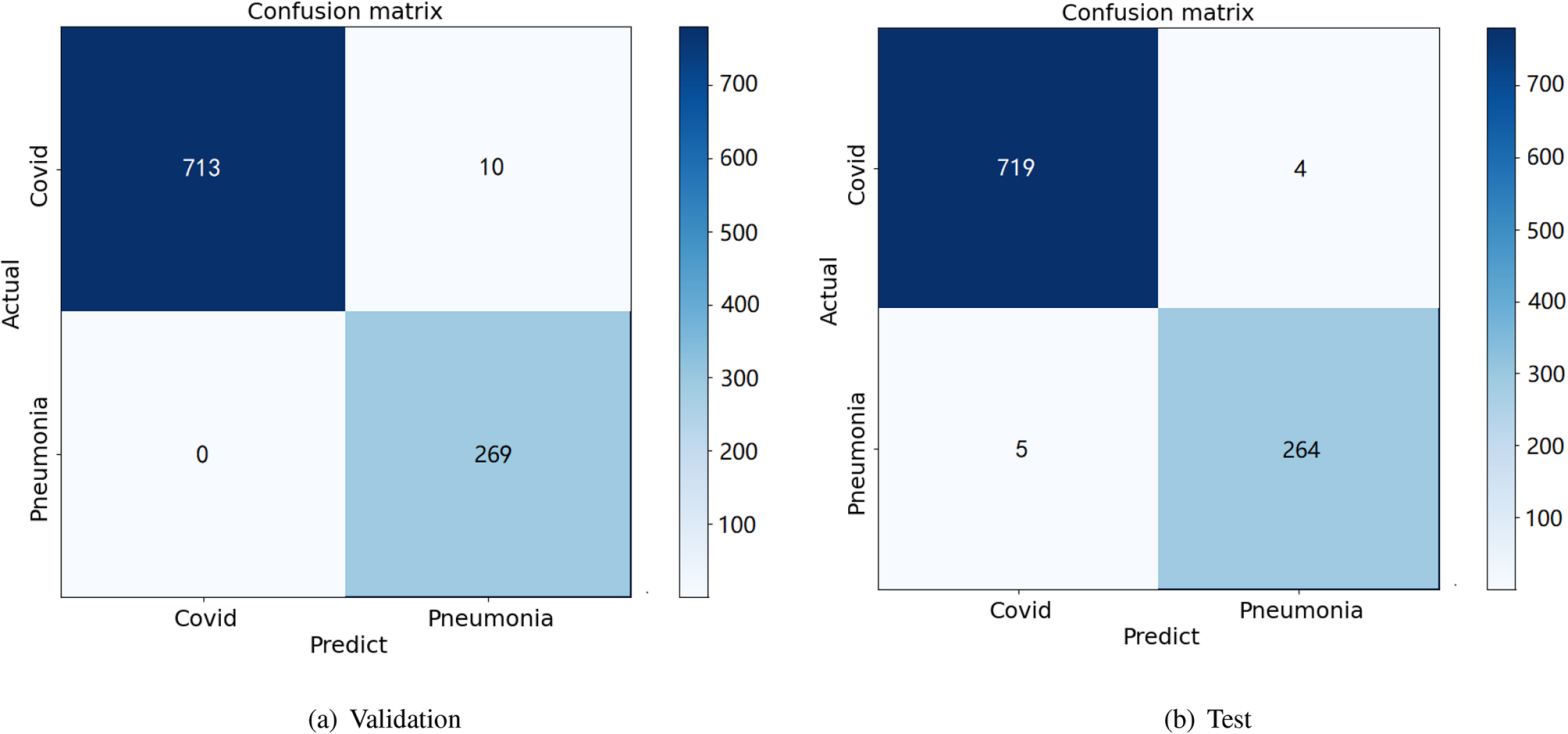
Confusion matrix for VGG16 on X-ray dataset: COVID-19/pneumonia.

**Figure 8:**
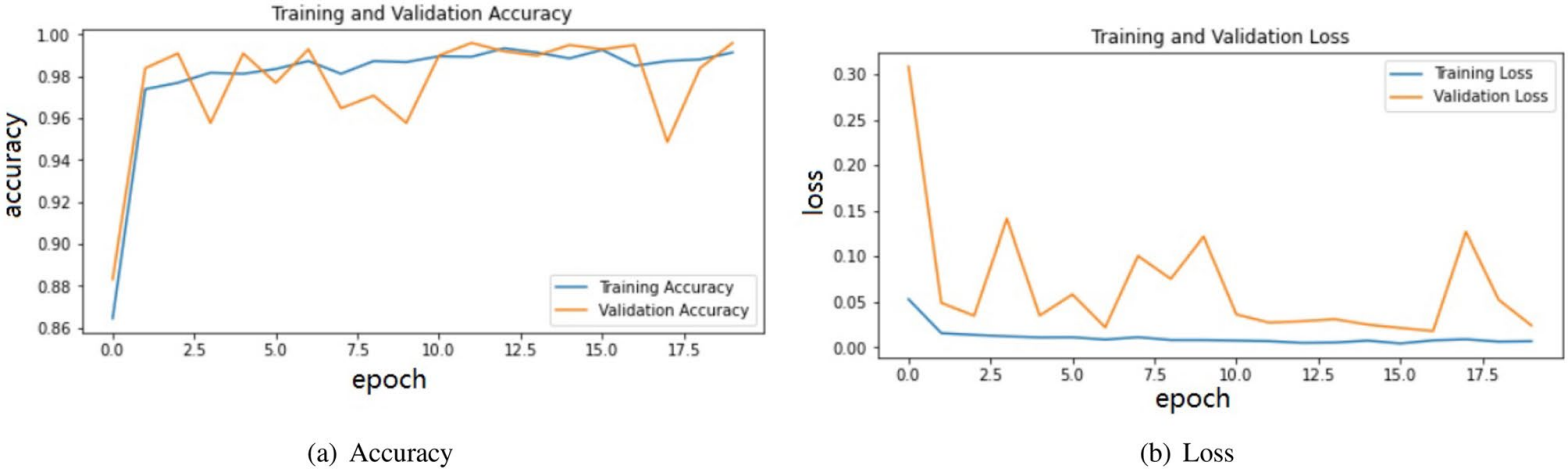
Model VGG16 for COVID-19/pneumonia on X-ray dataset.

Figure 7 shows the confusion matrix of validation and test data for two-class classification with COVID-19/pneumonia.As can be seen from it, this model also converges well. The Fig. 8 shows the training, validation accuracy and loss for the VGG16 model. From Fig. 8, the classification of COVID-19/pneumonia accuracy plot, after epoch 10, the accuracy starts to be stable where is equal to 99.5% for training data and validation data respectively. It is discovered that training and validation losses are similar to each other. The model fits well and avoid over-fitting.

### X-ray images results for three-class classification

To better validate our model, we conducted the three-class classification experiment. Table 3 summarises the results of our model for the three-class scenario of COVID-19/pneumonia/normal with the validation and test data. We provide the macro average scores for the multi-class experiments to indicate the overall performance across the different classes of the validation and test.

We can observe that our proposed model achieves promising results in the three-class classification test data with an accuracy of 97%. Moreover, from the validation data, the model achieves the high precision of 99% for the COVID-19 class and also has a high precision for the other class in the test data.

**Figure 9:**
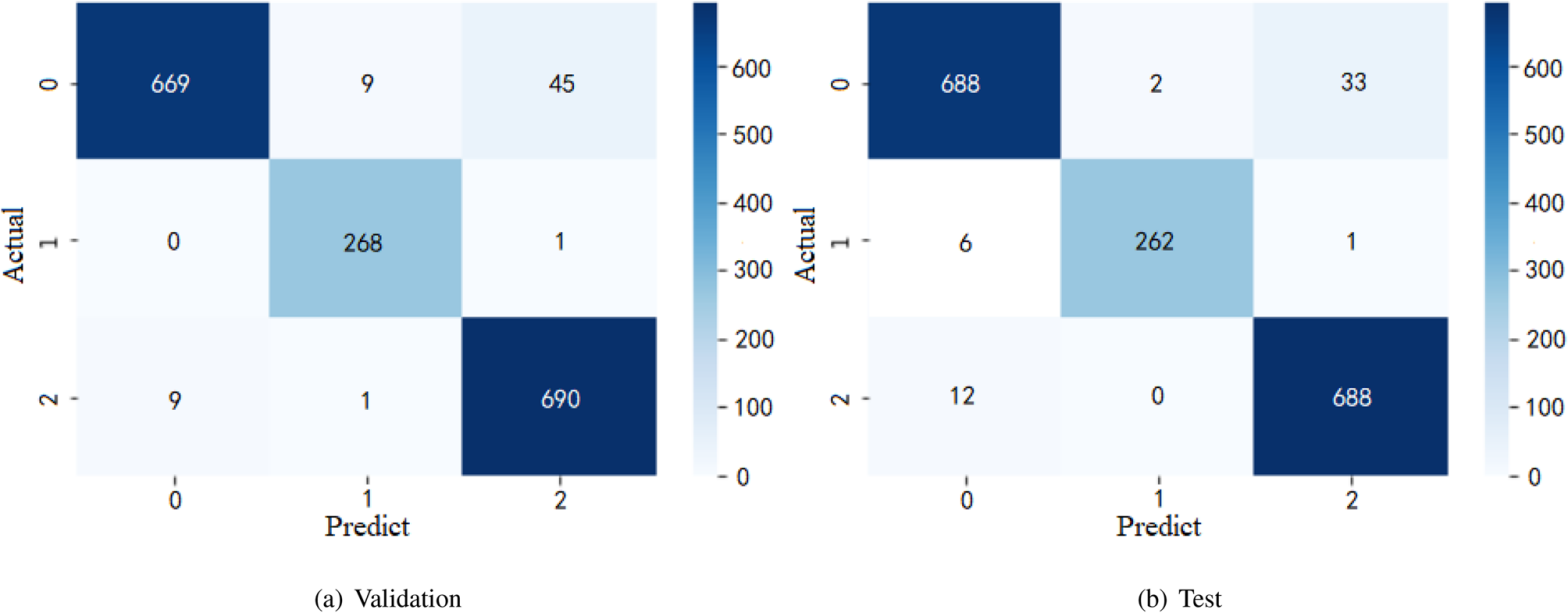
Confusion matrix for three-class classification on X-ray dataset: (Classes: 0—COVID, 1—Pneumonia and 2—Normal).

Figure 9 shows the confusion matrix for three-class classification of validation and test data. The covid and pneumonia classes achieve high precision values from the validation and test data, respectively. It indicates that out of all the classes, correct positive predictions could be classed as positive. However, some COVID-19 images were misclassified into the Normal category in both experiments. We analyzed these images that due to these patients are asymptomatic infections, their X-ray images are difficult to distinguish. To solve this problem, this type of image in the dataset should be increased and categorized for deep research.

**Table 3:** The performance for the validation and test data of three-class classification.

	Class	Precision (%)	Recall (%)	F1-score (%)	Accuracy (%)
Validation data	COVID	99	93	96	
	Pneumonia	96	100	98	
	Normal	94	99	96	
	Macro average	96	97	97	96
Test data	COVID	97	95	96	
	Pneumonia	99	97	98	
	Normal	95	98	97	
	Macro average	97	97	97	97

### CT-scan images dataset result

To assess the classification of CT-scan images of COVID-19, first we run VGG16, DenseNet121,ResNet50, and ResNet152 models with the dataset divided in training and validation sets at a ratio of 70:30. Fast.AI framework was then utilized in combination with ResNet model. Finally, we compared the performance obtained without Fast.AI and with Fast.AI.

#### CNN models without Fast.AI

Table 4 shows the results of applying the CNN models to scan CT images without using the Fast.AI framework. VGG16 provided the highest precision, 92%. It indicates the VGG16 model recognized the most COVID-19 CT-scan images out of all the positives. In terms of accuracy, recall and F1-score, the DenseNet121 model outperforms other models by 83.7%, 98.2% and 86.7%, respectively.

**Table 4:** Results of CNN models without Fast.AI.

Model	Accuracy (%)	Precision (%)	Recall (%)	F1-score (%)
VGG16	77	**92**	60	73
DenseNet121	**83**.**7**	77.7	**98**.**2**	**86.7**
ResNet50	81	84	77	80
ResNet152	80	89	70	78

Bold is used to show the maximum achieved for each parameter.

#### CNN models with Fast.AI

We then run the CNN models with Fast.AI for CT-scan images Classification. Fast.AI framework provides a very convenient high-level interface for transfer learning and built in a data set, therefore, the code to invoke the experiments can be very simple. The Interpretation function provided by Fast.AI lists some sample images that are identified incorrectly. We adopted the early-stopping strategy for hyper-parameter tuning to avoid the model being trained indefinitely, which wastes computing resources and degrades performance. Fast.AI saves each hyper-training parameter’s results and then returns the set of data before over-fitting occurs. The results of the experiments are summarized in Table 5. From this experiment, we can conclude that Fast.AI ResNet model achieves accuracy and F1-score both above 96%. Among them, Fast.AI ResNet50 achieves the highest accuracy, precision and F1-score of 96.3%, 97.6% and 96.4% respectively.

**Table 5:** Results of CNN models with Fast.AI.

Model	Accuracy (%)	Precision (%)	Recall (%)	F1-score (%)
Fast.AI ResNet50	96.3	97.6	95.2	96.4
Fast.AI ResNet152	96.2	95.7	97.2	96.4

**Figure 10:**
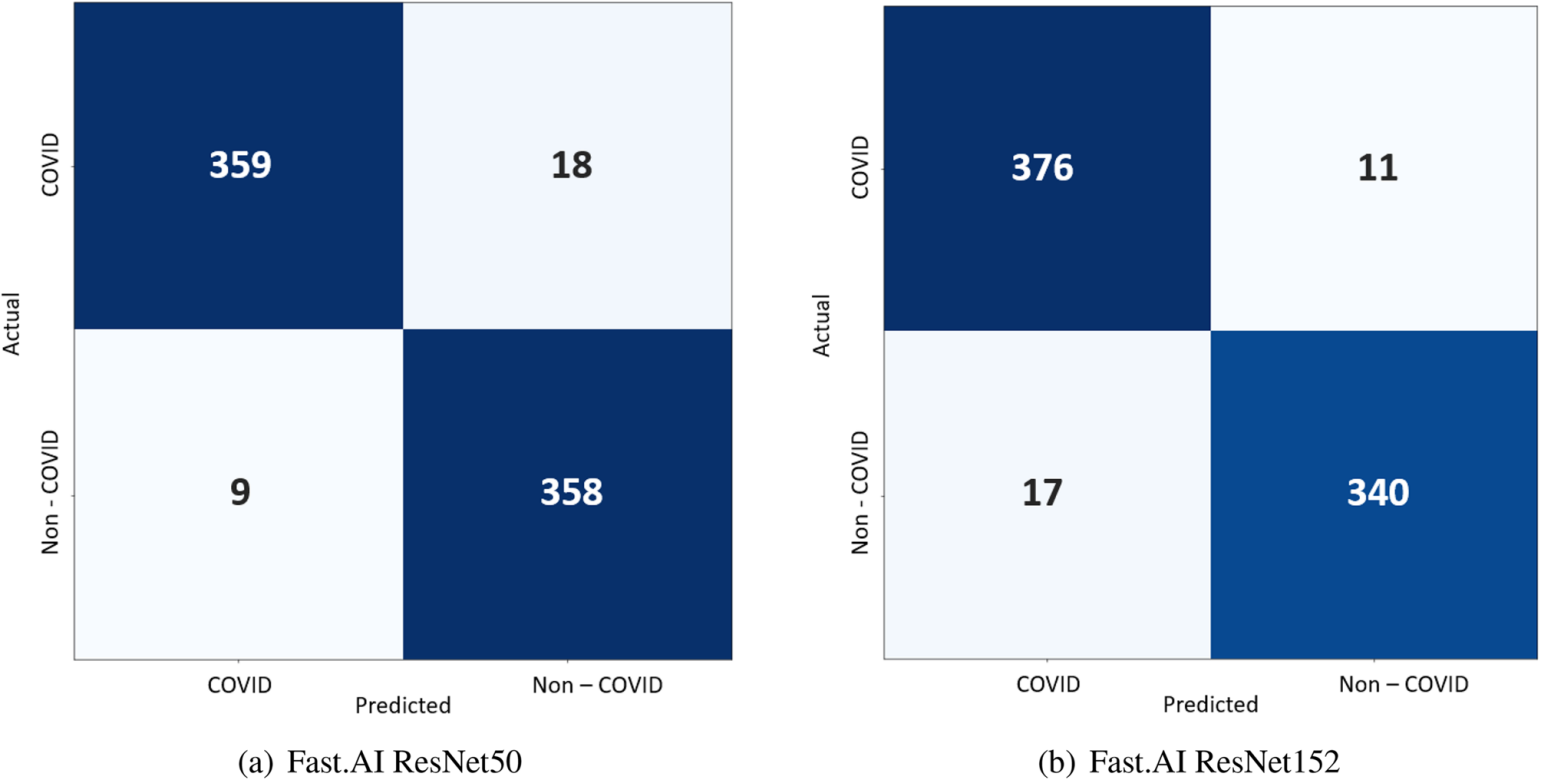
Confusion matrix for Fast.AI ResNet on CT-scan dataset.

**Figure 11:**
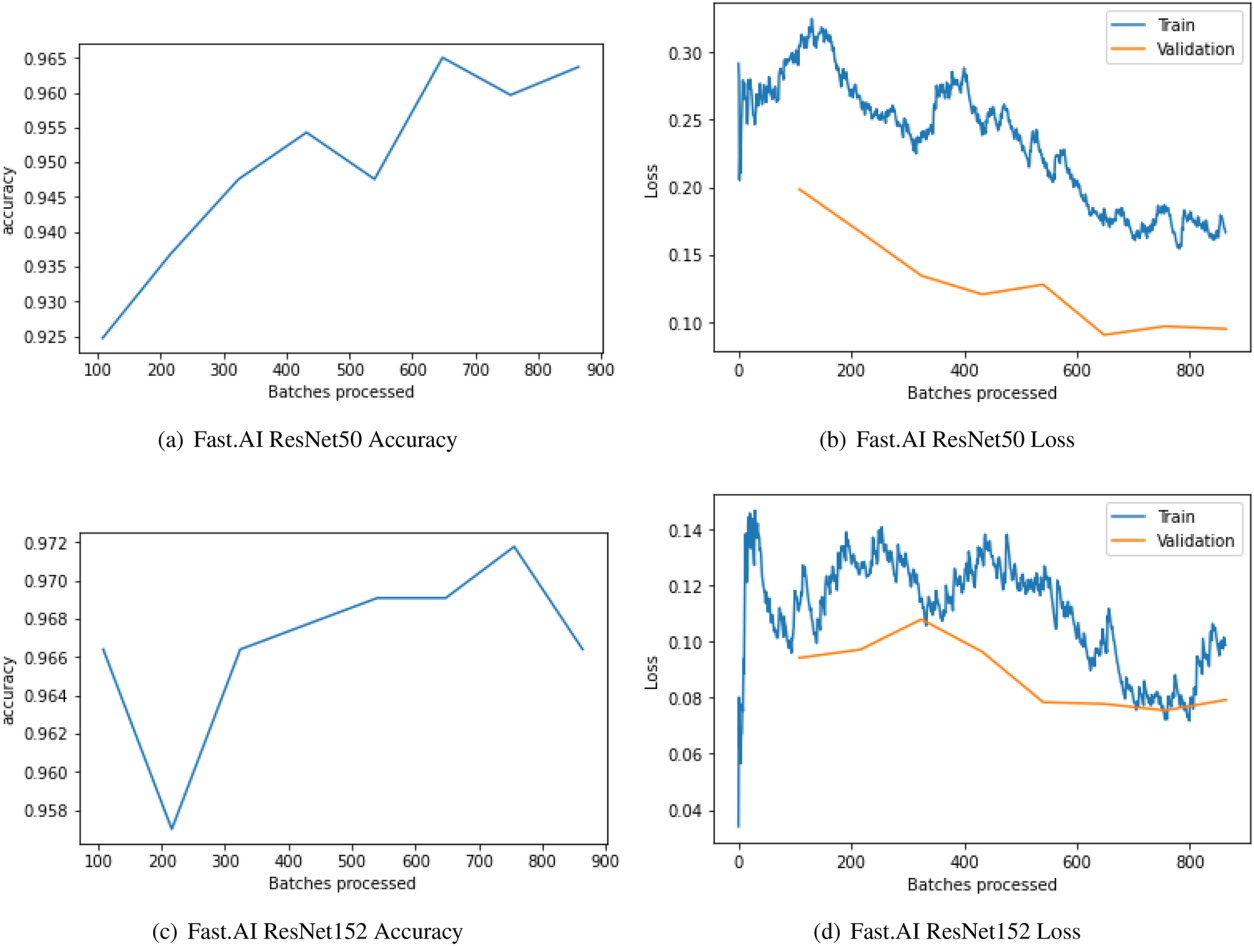
Model Fast.AI ResNet50 and Fast.AI ResNet152 for COVID/non-COVID on CT-scan dataset.

Figure 10 shows the confusion matrix for Fast.AI ResNet50 and Fast.AI ResNet152. we can observe that 359 images are classified as COVID-Positive and 358 images are classified as COVID-Negative correctly by Fast.AI ResNet50 model. Hence, our model correctly classified 717 images while 27 images are not correctly classified. Out of these 27 misclassified images, 9 images are predicted as COVID-Positive even though they were COVID-Negative, hence suffering from Type I error. Lastly, 18 images suffered from Type II error, where the images actually being COVID-Positive are predicted as COVID-Negative. Figure 11 shows the accuracy and loss plot of the Fast.AI ResNet50 (above) and Fast.AI ResNet152 (below) for COVID/Non-COVID on CT-scan dataset.

We can observe that the proposed models perform better after using Fast.AI than before using Fast.AI. Thus, we propose to use FAST.AI in conjunction with any of the ResNet models.

## Discussion

X-ray images are one of the typical imaging modalities used for COVID-19 research. For COVID-19 identification, CT-scans images provide high quality 3D images for COVID-19 detection. There are several research works for diagnosing COVID-19, with binary or multiple classifications, using chest X-rays. Some works use raw data and others have a feature extraction process. There are also differences in the number of images used in the research. The most preferred approach of the studies is the convolutional neural network (CNN). The classification performance of various CNN models can be checked in subsequent studies, by increasing the number of COVID-19 Chest X-ray images in the dataset. Table 6 compares several recent research works used in detection and analysis of COVID-19 with our proposed CNN models.

In the experiment of detection COVID-19 of X-ray images, our proposed model enhanced VGG16 has a better accuracy performance and a larger dataset than those in the previous studies. From Table 6, RG^28^ provides the best accuracy. They proposed a GDCNN frame where the training of each chest X-ray image generated by DCNN is fixed to 100 epochs, thus the proposed GDCNN has high computational and space complexity due to storing and evaluating a huge amount of DCNN structures. Our VGG16 model only needs to train 20 epochs, and the network structure is not so complicated. Shalbaf^29^ is different as they propose to use an assemble of 5 CNN and a majority vote at the end to classify CT images.The computation needs more time and is much higher, as they have to execute the 5 CNN methods for a single image. Rahimzadeh^30^ proposes an architecture ResNet50V2 as the backbone applies the feature pyramid network on CT-scan images. Though the model achieves 98% accuracy on more than 7996 test images, the COVID-19 precision is only around 81%. The number of COVID-19 images in the test is much lower than normal test images. They have 450 COVID-19 images and 7800 normal images for testing the network performance. The size of the images numbers differs greatly between the two categories. We use almost the same grade of images in the classification.

**Table 6:** Deep learning methods and techniques used in COVID-19.

Ref.	Model	Data type	ACC	F1	SN	SP
Khan^31^	CB-STM-RENet based on deep CNN	X-ray images,15127 images	0.97	0.95	-	-
Shalbaf^29^	The majority voting of 5 deep transfer learning architecture (EfficientNetB0, EfficientNetB3, EfficientNetB5, Inception_resnet_v2, Xception)	CT images, 349 CT images labeled positive for COVID-19 from 216 patient cases, 397 negative COVID-19 CT images as normal or contain other types of lung diseases from 171 cases.	0.85	-	0.85	-
RG^28^	Genetic Deep Learning convolutional neural network (GDCNN).	X-ray images, a total of more than 5000 CXR image, classifying pneumonia, normal and other pneumonia diseases	0.99	0.96	1	0.97
Sakib^32^	DL-CRC (CNN, GAN, generic)	X-ray images, three-class classification included 5794 pneumonia, 27228 normal images, 209 COVID-19	0.94	-	-	-
Jaiswal^33^	DenseNet201 based transfer learning	CT images, a total of 2492 CT-scans, 1262 are positive for COVID-19 and 1230 are negative	0.96	0.96	0.96	0.96
Rahimzadeh^30^	ResNet50V2 applies the feature pyramid network,designed layers	CT images,7996 images	0.98	-	-	-
Alshazly^14^	ResNet50	CT images, a total of 4173 images 2168 COVID-19 images	0.94	0.96	0.94	0.98
Ismael^34^	CNN combined with SVM classifier	X-ray images 200 Normal,2066 Pneumonia 116 COVID-19	0.94	0.94	0.91	0.99
Gomes^35^	Textures and shapes combined with SVM classifier	X-ray images 1583 Normal, 1490 V. Pneumonia 2783 B. Pneumonia 116 COVID-19	0.89	-	0.89	0.99
Majeed^36^	CNN network with 4 parallel layers and 16 filters	X-ray images 1575 Normal, 1346 V. Pneumonia 2529 Bact. Pneumonia 184 COVID-19	-	-	0.93	0.98
Misra^37^	Ensemble 3 ResNet-18	X-ray images three-class classification 1579 Normal, 4245 Pneumonia 184 COVID-19	0.94	-	1	-
Ozturk^38^	DarkCovidNet	X-ray images 125 COVID-19,500 No-findings	0.98	0.97	0.95	0.95

In comparison with the previous systems, we evaluated the model on two setups and with the three largest public datasets. Table 7 shows the best results of our system for X-ray and CT scan images. as may seen they are better than most of the approaches presented.

**Table 7:** Best result of our models for X-ray and CT scan images.

Task	Model	ACC	F1
X-ray COVID-19/normal	VGG16	0.98	0.985
X-ray COVID-19/pneumonia	VGG16	0.99	0.99
X-ray for three-class	VGG16	0.97	0.97
CT-scan with Fast.AI	ResNet50	0.963	0.964

Viral and bacterial Pneumonia symptoms are similar to COVID-19, thus its automatic classification would be very helpful to promote the screening process in clinical practice. In our work, we used deep learning approach to extract radiological features between COVID-19 and typical viral pneumonia. It is remarkable that our model has a very high accuracy (99%) distinguishing Pneumonia, particularly viral pneumonia, from COVID-19. In our opinion, more clinical data are needed to further validate and improve the validity of the model. The earlier the severe cases are detected, the more likely the treatment can be effective.

Moreover, the average value of testing accuracy with enhanced VGG16 model of binary classification is 98.5%, the average values of precision, recall and F1-score are 98.5%, 99% and 98.8% respectively. The value of testing accuracy, precision, recall and F1-score are all above 97% for the three-class classification task. Thus, the results prove that the enhanced VGG16 model has very promising results.

Our experimental results reveal the validity of our proposed networks, as they can achieve extremely promising results in binary classification tasks, with an average accuracy above 98% in X-ray images and 96.4% in CT-scan images. Our improved VGG16 and Fast.AI ResNet models have shown to be effective in COVID-19 patients from other pneumonia and healthy individuals. The proposed approaches favor the detection of false positives and false negatives and thus contributes to improve accuracy.

In conclusion, in our opinion the approach of combining deep learning and machine learning has substantially advanced and it can be a useful instrument for clinical practitioners and radiologists to facilitate the diagnoses and COVID-19 cases.

## Conclusion

The main goal of this paper was to research and discuss different Deep learning techniques applied to medical images for the diagnosis of COVID-19. We have created several datasets, from public repositories, including X-Ray and CT-Scan images for multi-class and binary class classification tasks. We have also validated VGG16 and ResNet deep learning structure models, for the classification of COVID-19 chest X-ray and CT-scan images. Training stage allowed us to adjust the models to establish a higher degree of accuracy as compared to previous works, as the accuracy of the enhanced CNN models is always above 98% and the confusion matrices show very few false cases for binary classification of X-ray images. The results demonstrate that the features derived from the enhanced deep learning models could be integrated into our work to build an effective model.

One of the significant findings in this paper is that with more public databases, data fusion models can further increase diagnostic and predictive performance. The other is that our models could effectively assist the virologists to diagnose COVID-19 and help the radiologists in the struggle against the outbreak of COVID-19, arriving in the diagnosis of critical patients in few minutes, which could be very important in their treatment.

As future research lines, we are already working on multi-criteria classification to distinguish images from datasets mixing patients with lung problems due to several possible diseases, such as tuberculosis, AIDS, COVID-19, etc. Moreover, we have not found datasets with metadata including stages of the disease to diagnostic the severity of the symptoms. We plan to work in this aspect in cooperation with doctors at some hospitals in Madrid.

## Data Availability

The used datasets were obtained from publicly open-source datasets from: COVID-19 Radiography Database https://www.kaggle.com/tawsifurrahman/covid19-radiography-database; OCT and Chest X-Ray Images https://data.mendeley.com/datasets/rscbjbr9sj/3; SARS-CoV-2 CT Scan Dataset https://www.kaggle.com/plameneduardo/sarscov2-ctscan-dataset.
